# IDH1 R132H Mutation Enhances Cell Migration by Activating AKT-mTOR Signaling Pathway, but Sensitizes Cells to 5-FU Treatment as NADPH and GSH Are Reduced

**DOI:** 10.1371/journal.pone.0169038

**Published:** 2017-01-04

**Authors:** Huixia Zhu, Ye Zhang, Jianfeng Chen, Jiangdong Qiu, Keting Huang, Mindan Wu, Chunlin Xia

**Affiliations:** 1 Soochow University, Soochow, Jiangsu, China; 2 Medical College, Nantong University, Nantong, Jiangsu, China; 3 Chinese medicine hospital, Wuxi, Jiangsu, China; State University of New York, UNITED STATES

## Abstract

**Aim of study:**

Mutations of isocitrate dehydrogenase 1 and 2 (IDH1 and IDH2) gene were recently discovered in vast majority of World Health Organization (WHO) grade II/III gliomas. This study is to understand the effects of IDH1 R132H mutation in gliomagenesis and to develop new strategies to treat glioma with IDH1 R132H mutation.

**Materials and methods:**

Over expression of IDH1 R132H in U87MG cells was done by transfecting cells with IDH1 R132H plasmid. MTT assay, scratch repair assay and western blot were performed to study effects of IDH1 R132H mutation on cell proliferation, migration, regulating AKT-mTOR signaling pathway and cell death respectively. NADP+/NADPH and GSH quantification assays were performed to evaluate effects of IDH1 R132H mutation on the production of antioxidant NADPH and GSH.

**Results:**

We found that over expression of IDH1 R132H mutation decreased cell proliferation consistent with previous reports; however, it increased cell migration and enhanced AKT-mTOR signaling pathway activation. Mutations in isocitrate dehydrogenase (IDH) 1 also change the function of the enzymes and cause them to produce 2-hydroxyglutarate and not produce NADPH. We tested the level of NADPH and GSH and demonstrated that IDH1 R132H mutant stable cells had significantly low NADPH and GSH level compared to control or IDH1 wild type stable cells. The reduced antioxidants (NADPH and GSH) sensitized U87MG cells with IDH R132H mutant to 5-FU treatment.

**Conclusion:**

Our study highlights the important role of IHD1 R132H mutant in up- regulating AKT-mTOR signaling pathway and enhancing cell migration. Furthermore, we demonstrate that IDH1 R132H mutation affects cellular redox status and sensitizes gliomas cells with IDH1 R132H mutation to 5FU treatment.

## Introduction

Gliomas make up about 80% of all malignant brain tumors.[1] The exact causes of gliomas are not well known and it is believed that several oncogenes cooperate and contribute to the development of gliomas. [2] It was found that either isocitrate dehydrogenase (IDH) 1 or 2 genes mutations frequently occur in gliomas. [3] Isocitrate dehydrogenase (IDH) enzyme catalyzes the oxidative decarboxylation of isocitrate to produce α-ketoglutartate and at the same time use NADP+ as a cofactor to generate NADPH and maintain cellular redox status.[4] IDH1 mutations occurred in vast majority of World Health Organization (WHO) grade II/III gliomas and secondary glioblastomas. [5] Mutations in IDH1 occur only at specific arginine residues in the active sites of the enzymes and the most common mutation is R132H, which composes more than 80% of all IDH mutations. [5–7] The R132H mutation confers a gain-of-function activity that reduces α-ketoglutarate (-α- KG) to produce D-2-hydroxyglutarate (D2HG) and at the same time consumes NADPH. [8] The effects of IDH1 R132H mutation causes widespread metabolic changes including decreased levels of glutathione metabolite and increased glutaminolysis in order to maintain normal levels of key TCA cycle metabolites. [9–11] The depletion of α- KG caused by IDH mutations in human tumor causes deregulation of multiple α-KG-dependent dioxygenases, which are involved in the hydroxylation of various protein, histones, transcription factors and alkylated DNA and RNA. [12–16] Due to such a broad spectrum of substrates of α-KG-dependent dioxyneases, IDH1 mutation is expected to potentially affect multiple cellular pathways.

Bralten, L. B. et al. found that IDH1 R132H mutation in U87 cell line significantly decreased cell proliferation, accompanying changes in cell morphology and cell migration patterns. [17] In addition, Sabit, H. reported that the levels of mutation of IDH1 R132H occurring increased with higher grade of glioma in clinical specimens of glioma. [18] Malignant tumor cells are known to have high proliferating rate, and has anti-apoptotic and immortalized malignant phenotype which results in rapid progression. Malignant glioma cells are particularly well known by their aggressively invasive ability. Glioma tumor cells without capsule can invade the surrounding normal tissue and lead to difficulties in completely resecting gliomas by surgery. We are still at the infancy stage of understanding the role of IDH1 and IDH1 R132H mutation in gliomagenesis and further in-depth understanding of its molecular mechanisms in regulating cell proliferation and migration will be critical to develop future targeted therapy. Therefore, we used multiple approaches to investigate the role of IDH1 and IDH1 R132H mutant in affecting cell proliferation, migration and major cell signaling pathway AKT-mTOR by stably overexpressing IDH1 either wild type or R132H mutant in U87MG cells or knocking down IDH1 by siRNA.

We further extend our study to explore future treatment options for IDH1 mutated tumor. Fonnet et al found that in glioblastoma tumor samples occurrence of IDH1 R132H mutation reduced this capacity to produce NADPH by 38% and furthermore mutated IDH1 consumes rather than produces NADPH. [19] Therefore, NADPH production is hampered in glioblastoma with IDH1 R132H mutation. This provides therapeutic opportunities to exploit the metabolic vulnerabilities specific to IDH1 mutated tumor. Concurrently it was reported that patients with IDH mutated glioblastoma has prolonged survival. [20, 21] It is highly possible that the low NADPH levels may sensitize glioblastoma to irradiation and chemotherapy. Therefore, we tested the level of NADPH and reduced glutathione (GSH) in U87MG cells overexpressing IDH1 R132H mutation and its sensitivity to chemotherapy drug 5-FU compared to its wild type or vector control.

## Methods and Materials

### Cell lines

U87MG cells were purchased from ATCC. Cells were maintained in Dulbecco's Modified Eagle Medium (DMEM) (HyClone, Logan, UT) containing 10% fetal bovine serum (FBS) supplemented with penicillin (100 U/mL) and streptomycin (100 mg/mL). These cells were cultured in accordance with the suppliers’ instructions.

### Transfection and stable clone selection

The IDH1-Flag and IDH1 R132H-Flag plasmids constructed into pCMV-Tag2B vector were purchased from Addgene (Addgene Company, Cambridge, MA). The transfection was done using LipofectamineTM 2000 from Invitrogen and stably transfected cells were selected using G418 and tested using anti-Flag antibody by western blotting.

### SiRNA knocking down of IDH1

Control SiRNA (sc-37007), siRNA targeting IDH1 (sc-60829), siRNA transfection reagent (sc-29528) and siRNA transfection medium (sc-36868) were purchased from Santa Cruz (USA). Briefly, U87 cells were plated and transfected with either SiRNA control or siRNA targeting IDH1 using siRNA transfection reagent (sc-29528) following the protocol from the manufacture. After 2 days, cells were harvested and protein levels of IDH1 were detected using western blots.

### 3-(4,5-cimethylthiazol-2-yl)-2,5-diphenyl tetrazolium bromide (MTT) cytotoxicity assay

The U87MG cells and its stable cells over expression of IDH1 or IDH1 R132H mutant were plated at same cell number and cultured in 96-well flat-bottomed microtiter plates supplemented with DMEM containing 10% fetal bovine serum and kept in a humidified incubator containing 95% air and 5% CO2 at 37°C. After 24 hours, viable cell number was determined by MTT assay at 570nm.

### Western blotting

Cells were rinsed once with PBS and lysed by using sodium dodecylsulfate (SDS) sample buffer. Equal amounts of protein from each group were separated on 10% SDS-polyacrylamide gels. Proteins were transferred to Immobilon P membranes (Millipore). The membranes were blocked with 5% non-fat dry milk in Tris-buffered saline for one hour at room temperature and incubated with the appropriate antibody in TBS-T containing 5% non-fat dry milk overnight at 4°C. After washing in TBS-T the membrane was incubated with the appropriate horseradish peroxidase (HRP)-conjugated secondary antibody. Proteins were detected using the chemiluminescent substrate. Anti-Flag antibody (cat #LT0420, Life Tein), p-mTOR site of phosphorylation (cat# ab109268, Abcam), mTOR (cat# ab32028, Abcam), p-AKT site of phosphorylation (cat# 4061, CST), AKT (cat# 4691, CST), GAPDH (cat# ab181602, Abcam), caspase-3 (cat# AC030, Beyotime), cleaved caspase 3 (cat#AC033, Beyotime).

### Scratch repair assay

The cells were plated at 1.5×105 cells per well in a 12-well plate and grown overnight in incubator at 37°C with 5% CO2. After one day, a straight line scratch was made on a confluent monolayer of cells using a sterile 1 ml disposable serological pipette. Then the cells were washed with 1 ml DMEM to remove debris. Photos were taken at 0, 24, and 48 h after adding drug. The distance of migration and migration index were calculated by Image-Pro Plus 6.0 software (Media Cybernetics Inc., Rockville, MD, USA) and the following formula: inhibition of cell migration (%) = (1 –migration distance of the experimental group/migration distance of the control group) × 100%.

### Quantification of GSH

GSH and GSSG were quantified using a GSH-Glo™ Glutathione Assay Kit (V6912, Promega) according to the manufacturers’ instructions, following incubation of 1×10^4^ cells/well in 96-well plates incubated for one hour at 37°C with 5% CO2 in serum-free HBSS with added Ca2+ and Mg2+.

### Quantification of NADP+/NADPH

Cells (4×10^6^ per well) were incubated for one hour at 37°C with 5% CO2 in HBSS. Wash cells with cold PBS. Pellet cells in a tube by spinning at low speed for 5 minutes, and discard supernatant. NADPH was quantified using a NADP+/NADPH assay kit (ab65349, Abcam), according to the manufacturers’ instructions.

### Statistical analysis

Error bars shown are standard deviations from the mean of at least three replicates. Two-tailed pairwise Student’s t tests were used to compare two groups. P values less than or equal to 0.05 were considered to have significance.

## Results

### Establishment of stable cells overexpressing IDH1 and IDH1 R132H mutant

**Fig 1:**
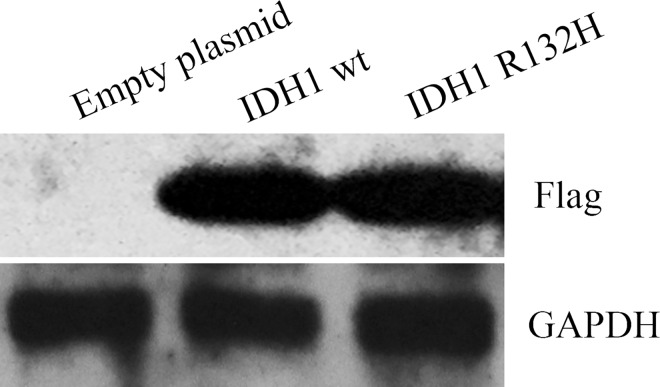
Establishment of stable cells overexpressing IDH1 wild type and IDH1 R132H mutant. Expression of IDH1 in U87MG vector control stable cells, IDH1 wild type stable cells and IDH1 R132H stable cells were test by western blotting with anti-Flag antibody.

In order to understand the effects of IDH1 wild type and IDH1 R132H mutant on cell proliferation and migration, U87MG stable cells over-expressing either empty vector or pCMVtag-2B containing IDH1 wild type or IDH1 R132H mutant were established. Fig 1 demonstrated that the over expression of IDH1 wild type and IDH1 R132 mutant in selected stable cells compared to vector control U87MG cells.

### Over expression of IDH1 R132H mutant but not IDH1 wild type decreases cell proliferation

**Fig 2:**
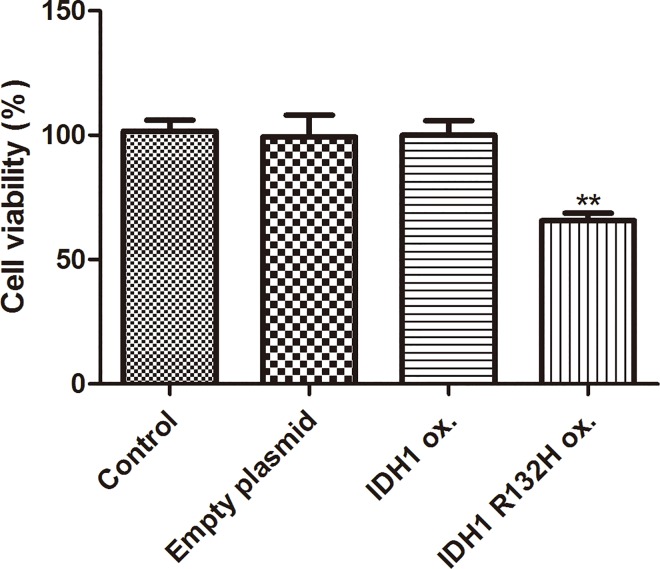
Over expression of IDH1 R132H mutant but not IDH1 wild type decreased cell proliferation. The rate of proliferation of parental U87MG, vector control stable cells, IDH1 wild type and IDH1 R132H stable cells were examined using MTT assay. IDH1 R132H mutant stable cells vs. parental control, vs. empty vector stable cells. (p<0.05).

Rate of proliferation of stable cells overexpressing empty vector, IDH1 wild type or IDH1 R132H mutant was performed using MTT assay. Over expression of IDH1 wild type did not affect cell proliferation compared with parental control cells and empty vector control stable cells. However, IDH1 R132H mutant stable cells significant decreased cell proliferation compared to the parental control and empty vector stable cells (Fig 2). This suggested that over expression of IDH1 R132H mutant but not IDH1 wild type decreased cell proliferation.

### Over expression of IDH1 R132H mutant but not IDH1 wild type increases cell migration

**Fig 3:**
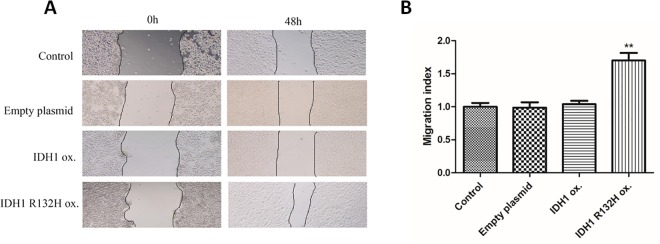
Over expression of IDH1 R132H mutant but not IDH1 wild type increase cell migration. A. Images of parental U87MG, vector control cells and stably transfected U87MG cells overexpressing IDH 1 wild type or IDH1 R132H mutant were taken at 0 and 48 hours after scratch assay. B. Migration index was used to quantify the cell migration. IDH1 R132H mutant stable cells vs. parental and vector control. (p<0.05).

Scratch repair assay was performed to evaluate the effects of IDH1 wild type and IDH1 R132H mutant on cell migration Images were taken at 0 and 48 hours after scratch was performed (Fig 3A). Migration index was used to quantify the cell migration as shown in Fig 3B. Over expression of IDH1 wild type did not affect cell migration compared with parental control cells and empty vector control stable cells. However, IDH1 R132H mutant stable cells significant increased cell migration compared to the parental control and empty vector stable cells (Fig 3A and 3B). This suggested that over expression of IDH1 R132H mutant but not IDH1 wild type increased cell migration.

**Fig 4:**
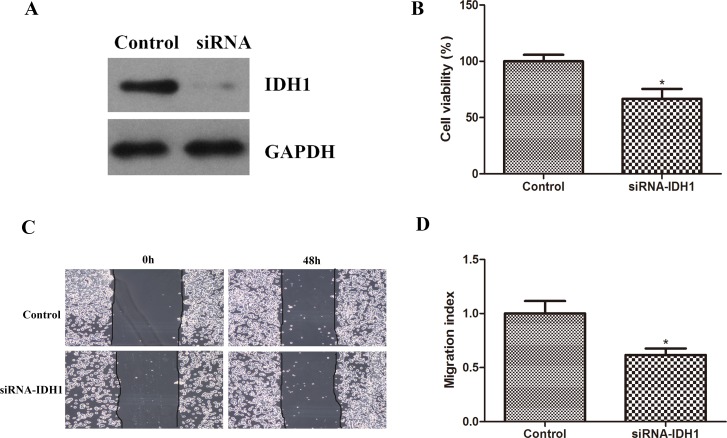
siRNA IDH1 decreased cell proliferation and migration. A. Western blotting performed to detect levels of IDH1 in control and SiRNA-IDH1 cells. B. The rate of proliferation of parental U87MG, siRNA-IDH1 cells. (p<0.05). C. Images of parental U87MG, siRNA-IDH1 cells taken at 0 and 48 hours after scratch assay. D. Migration index was used to quantify the cell migration. Control cells vs.siRNA-IDH1 cells. (p<0.05).

Zhao. S et al found that IDH1 mutants can form dimers with the wild type IDH1and further inhibits IDH1 enzyme activity. [22] In order to understand whether over expression of IDH1 R132H mutant confer similar effects as inhibiting IDH1 activity on cell proliferation and cell migration, we used SiRNA to knock down IDH1 and examine its effects on cell proliferation and migration. We found that knocking down of IDH1 using SiRNA decreased cell proliferation and migration as shown in Fig 4. The inhibiting of IDH1 enzyme activity by IDH1 R132H mutants or knocking down its protein by siRNA had similar inhibitory effects on cell proliferation but different effects on cell migration. This raises the question that IDH1 R132H mutant has unique character on cell migration different from its effects on wild type IDH1 enzyme activity. AKT-mTOR signaling pathway is the major signaling pathway regulating cell proliferation and migration. Therefore, we decided to examine the effects of over expressing IDH1 and IDH1 R132H or knocking down IDH1 on AKT-mTOR signaling pathway in an effort to understand their different role in regulating cell migration.

### IDH1 R132H mutant stable cells have increased activation of AKT-mTOR signaling pathway while knocking down of IDH1 inhibited activation of AKT-mTOR activation

**Fig 5:**
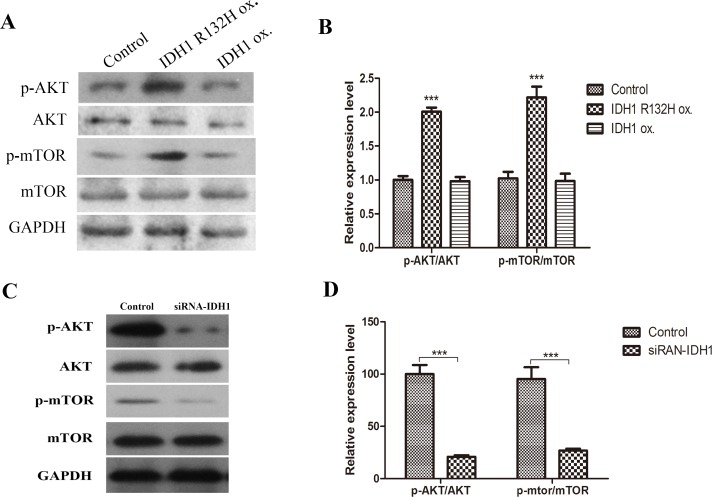
IDH1 R132H mutant stable cells have increased activation of AKT-mTOR signaling pathway, while knocking down IDH1 inhibited AKT-mTOR activation. A. Western blotting was performed to detect activated P-AKT and p-mTOR, total AKT and mTOR in stable cells overexpressing vector, IDH1 R132H mutant or IDH1 wild type. B. Quantification of p-AKT/total AKT, p-mTOR/total mTOR. p-AKT/total AKT and p-mTOR/total mTOR in R132H mutant stable cells vs. control or IDH1 wild type stable cells (p<0.05). C. P-AKT and p-mTOR, total AKT and mTOR were detected in control cells and cells transfected with siRNA targeting IDH1. D. Quantification of p-AKT/total AKT, p-mTOR/total mTOR. p-AKT/total AKT and p-mTOR/total mTOR in Control vs. SiRNA-IDH1 (p<0.05).

Western blotting was performed to detect activated p-AKT and p-mTOR. IDH1 R132H mutant stable cells had higher level of p-AKT and p-mTOR compared to control and IDH1 wild type stable cells as shown in Fig 5A and 5B was the quantification of p-AKT/total AKT, p-mTOR/total mTOR. There was significant increase of p-AKT/total AKT and p-mTOR/total mTOR in R132H mutant stable cells compared with control or IDH1 wild type stable cells. This suggests that overexpressing IDH1 R132H mutant activated AKT-mTOR signaling pathway.

Knocking down of IDH1 by siRNA demonstrated that there was decreased AKT-mTOR signaling pathway (Fig 5C and 5D), which was completely different from IDH1 over expression of R132H mutant. It provides evidence that even though IDH1 R132H has similar effects on decrease IDH1 enzyme activity and inhibiting cell proliferation similar to siRNA knocking down of IDH1. However, it has total different effects on AKT-mTOR signaling pathway regulation. IDH1 knocking down inhibits ATK-mTOR while IDH1 R132H enhances AKT-mTOR signaling pathway. AKT-mTOR signaling pathway activation played an important role in migration and invasion [23–26] by targeting eNOS and up-regulating ICAM, promoting migration via activation of p70S6K, increased matrix metalloproteinases, MMP and increased β-Catenin and decrease E-cadherin. The altered effects on AKT-mTOR signaling activation by siRNA-IDH1 and over expression of IDH1 R132H could be one of the mechanisms contributing to their different effects on cell migration as shown in Figs 3 and 4.

### IDH1 R132H mutant overexpressing sensitized cells to 5-FU treatment by enhancing apoptosis

**Fig 6:**
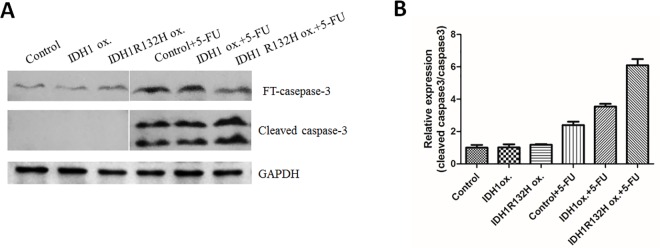
IDH1 R132H mutant overexpressing sensitized cells to 5-FU treatment by enhancing apoptosis. A. Control cells, IDH1 wild type stable cells, IDH1 R132H mutant stable cells were treated without or with 5-FU for one day. Cleaved caspase 3 was used to detect level of apoptosis. B. Ratio of cleaved caspase 3 to caspase 3 was quantified. (Control + 5FU) vs. (IDH1 R132H ox + 5FU) (p<0.05).

IDH1 R132H mutation increased AKT-mTOR signaling pathway, which could contribute to its oncogenic property. However, it is interesting that gliomas with mutated IDH1 have improved prognosis compared to gliomas with wild-type IDH. We decide to test their sensitivity to 5-FU treatment. Cleaved caspase 3 was used to detect level of apoptosis. IDH1 R132H mutant stable cells had the highest amount of cleaved caspase 3 detected after 5-FU treatment as shown in Fig 6A and 6B. This suggested that IDH1 R132H mutation is more vulnerable to 5-FU treatment even though they have increased cell migration and increased activation of AKT-mTOR.

### IDH1 R132H mutant stable cells have decreased NADPH and GSH

**Fig 7:**
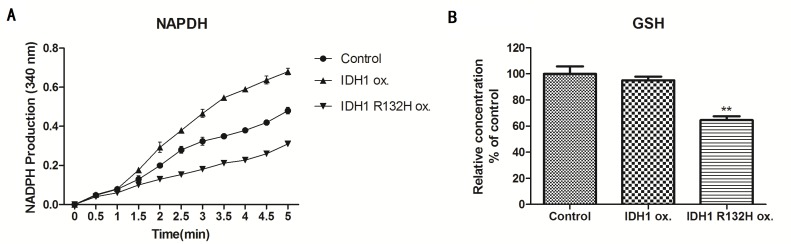
IDH1 R132H mutant stable cells have decreased NADPH and GSH. A. NDAPH level detected in control cells, IDH1 wild type stable cells and IDH1 R132H mutant stable cells. IDH1 R132H mutant vs. control (P<0.05). B. GSH level detected in control cells, IDH1 wild type stable cells and IDH1 R132H mutant stable cells. IDH1 R132H mutant vs. control vs. IDH1 wild type (p<0.05).

To further understand the mechanism of increased vulnerability to 5-FU treatment in IDH1 R132H mutant stable cells, we examined the level of NADPH and GSH which are the antioxidant to protect cells from damages caused by reactive oxygen species. Fig 7A demonstrated that overexpress wild type IDH1 increased NADPH level but overexpression of IDH1 R132H mutant decreased NADPH level. IDH1 R132H mutant stable cells also had significant decreased GSH level compared to control and wild type cells as shown in Fig 7B.

## Discussion

We established the stable cell lines over-expressing IDH1 wild type and IDH1 R132H mutant in U87 cells. Over expression of IDH1 did not have significant effects on cell proliferation and migration while IDH1 R132H mutation significantly decreased cell proliferation and increased cell migration. Our data are consistent with previous founding by Linda. B et al who have shown that IDH1 R132H overexpression in established glioma cell lines in vitro resulted in a marked decrease in proliferation. [17] Nie. Q et al also demonstrated that IDH1 R132H decreased the proliferation of U87 glioma cells through upregulation of microRNA-128a. [27]

Jin. G et al found that knocking down of IDH1 wild type or overexpressing IDH1 R132H mutant in HT1080 cells inhibited cell proliferation.[28] Zhao. S et al found that IDH1 mutants can form dimers with the wild type IDH1and inhibits IDH1 enzyme activity. [27] It is possible that over expression of IDH1 R132H inhibited IDH1 enzyme activity in U87 cells and lead to cell proliferation inhibition. In order to confirm the role of IDH1 in cell proliferation, even though over expression of IDH1 wild type did not have significant effects on cell proliferation, we decided to use siRNA to knock down of IDH1 and found that knocking down of IDH1 in U87 cells inhibited cell proliferation and migration. These provided evidence that down regulating IDH1 activity either by siRNA or overexpressing IDH1 R132H mutant would inhibit cell proliferation.

However, the inhibition of IDH1 using siRNA knocking down and over expressing IDH1 R132H have same effects on cell proliferation but total opposite effects on cell migration. It is highly possible that besides its effects on inhibiting IDH1 enzyme activity by over expression of IDH1 R132H, IDH1 R132H has other major different effects on factors involved in cell migration. Sabit, H. reported that in clinical specimens of glioma by the levels of mutation of IDH1 R132H occurring increased with higher grade of invasion of glioma. [23] It is consistent with our finding that IDH1 R132H promoted cell migration. Therefore, we looked at the signaling pathway involved in cell proliferation and migration by comparing vector control with either IDH1 wild type, IDH1 R132H mutant and siRNA IDH1 cells.

We found that IDH1-SiRNA decreased AKT-mTOR while IDH1 R132H significantly increased AKT-mTOR signaling activation. The different effects on AKT-mTOR may contribute to its different effects on cell migration. AKT as an oncogene has been shown to be activated in prostate, glioma and melanoma together with the loss of PTEN. The activated AKT further activates the downstream mTOR signaling pathway, which are involved in gene transcription, protein translation involved in cell survival and antiapopotosis. [29, 30] AKT-mTOR signaling pathway activation also played an important role in migration and invasion [23–26] by targeting eNOS and up-regulating ICAM, promoting migration via activation of p70S6K, increased matrix metalloproteinases, MMP and increased β-Catenin and decrease E-cadherin. Therefore, activation of AKT-mTOR signaling pathway played an important role in the enhanced migration by IDH1 over expression of R132H.IDH1-siRNA inhibited AKT-mTOR signaling pathway, therefore, prevented cell migration.

Our finding of the activation of AKT-mTOR signaling pathway by IDH1 R132H mutation could contribute to further understanding its effects on cell migration. It also explained why the IDH1-SiRNA decreased migration by inhibiting ATK-mTOR signaling pathway.

These provided important insight of the function of IDH1 and IDH1 R132H in regulation of cell proliferation and migration. It also provided targeted treatment options for IDH1 wild type and IDH1 R132H mutant tumor respectively. For glioma without IDH1 mutation, IDH1 can be targeted using siRNA or antagonist to inhibit enzyme activity and further down-regulating ATK-mTOR signaling and cell proliferation and migration. For glioma with IDH1 R132H mutant, which caused the activation of AKT-mTOR signaling pathway, targeting AKT-mTOR could be adopted to abolish its effects on migration and invasion. Future experiments will need to be done to explore the treatment options targeting AKT-mTOR in IDH1 R132H mutant glioma both in vitro and in animal glioma tumor models.

At the same time, we explore the application of 5-FU in treatment of IDH1 R132H mutant glioma stable cells. IDH1 R132H mutation confers a gain-of-function activity that reduces α- KG to produce D-2-hydroxyglutarate (D2HG) and consumes NADPH [12] at the same time. Several studies have demonstrated that IDH1 R132H mutation causes widespread metabolic changes including affecting DNA methylation and DNA repair. [31, 32] Fonnet et al found that in glioblastoma tumor samples occurrence of IDH1 R132 mutation reduced this capacity to produce NADPH by 38% and furthermore mutated IDH1 consumes rather than produces NADPH. [22] NADPH plays an important role in regulating redox status of cells, and prevents oxidative damages. Our data showed that IDH1 R132H mutation significantly decreased NADPH and GSH levels compared to vector control and IDH1 wild type stable cells, and this is associated with increased sensitivity to chemotherapy drug 5-FU. This further emphasized the important role of NADPH as antioxidant. When NADPH production is compromised by IDH1 R132H mutation, it actually conferred metabolic vulnerability for those IDH1 R132H mutated cells to chemotherapy drug. Further experiments will be done to explore choices of chemotherapy drugs or radiation therapy in this specific IDH1 R132H mutated cells. It is also possible to extend this study to other IDH mutations in the future to examine whether they have similar response as IDH1 R132H mutants.

In conclusion, our study demonstrates the role of IDH1 R132H mutation in gliomagenesis by activating AKT-mTOR signaling pathway and its pivotal role in promoting cell migration and invasion. The AKT-mTOR signaling pathway activation by IDH1 R132H will provide new evidence on future application of other combined targeted therapy for glioma with IDH1 R132H mutations. Furthermore, we confirmed the role of IDH1 R132H mutations in affecting cellular redox status using glioma cell lines, and provided evidence to further explore the application of chemotherapy such as 5-FU in the treatment of gliomas with IDH1 R132H mutation.
